# Underweight, overweight, and weight change in older family caregivers and their care recipients: longitudinal evidence from a randomized controlled trial

**DOI:** 10.3389/fragi.2024.1376825

**Published:** 2024-08-15

**Authors:** Sohvi Koponen, Irma Nykänen, Roosa-Maria Savela, Tarja Välimäki, Anna Liisa Suominen, Ursula Schwab

**Affiliations:** ^1^ Institute of Public Health and Clinical Nutrition, School of Medicine, University of Eastern Finland, Kuopio, Finland; ^2^ INVEST Research Flagship Centre, University of Turku, Turku, Finland; ^3^ Department of Nursing Science, University of Eastern Finland, Kuopio, Finland; ^4^ Oral and Maxillofacial Diseases Teaching Unit, Kuopio University Hospital, Kuopio, Finland; ^5^ Department of Medicine, Endocrinology and Clinical Nutrition, Kuopio University Hospital, Wellbeing Services County of North Savo, Kuopio, Finland

**Keywords:** caregiving, frailty, nutritional status, older people, overweight, underweight, weight loss

## Abstract

This study aimed to identify differences among body mass index (BMI) categories of older family caregivers (≥60 years) and their care recipients (≥65 years). Secondly, this study aimed to examine group differences and factors associated with weight change during a nutrition and oral health intervention. This secondary analysis of a randomized controlled trial (ClinicalTrial.gov (NCT04003493)) involved individually tailored nutritional guidance from a clinical nutritionist and oral health guidance from a dental hygienist. Baseline BMI differences were analyzed, followed by further analyses of group differences and associated factors of weight change over a 6-month period using generalized estimating equations. Among the participants (113 family caregivers and 107 care recipients), 36.3% and 35.1% were overweight (BMI >29 kg/m^2^), while 18.6% and 21.6% were underweight (BMI <24 kg/m^2^) at baseline, respectively. For family caregivers differences in BMI categories included age, mid-arm and calf circumferences, and plasma prealbumin concentration. For care recipients differences were observed in medication use, mid-arm and calf circumferences, Mini Nutritional Assessment scores, physical function, and number of teeth. During the 6-month intervention, there were no differences in weight change between intervention and control groups for both caregivers and care recipients. Factors significantly associated (*p* < 0.05) with weight loss included female sex for both caregivers and care recipients, and frailty for caregivers. Family caregivers’ characteristics were not significantly associated with weight change in their care recipients. In conclusion, being overweight is a prevalent among older family caregivers and care recipients. Factors such as age, medication use, physical function, number of teeth, and Mini Nutritional Assessment scores varied across BMI categories. Female sex was associated with weight loss in both older family caregivers and care recipients, and frailty was associated with weight loss in caregivers. However, the characteristics of family caregivers did not explain the weight loss of their care recipients.

Clinical Trial Registration: [https://www.ClinicalTrial.gov/], identifier [NCT04003493].

## 1 Introduction

The aging population in Finland needs growing attention, as individuals aged 60 and above constitute nearly one-third of the total population (World Health Organization, 2023). This demographic faces many health challenges, including multimorbidity (Yarnall et al., 2017), cognitive decline (Langa and Levine, 2014), increased risk of malnutrition (Tombini et al., 2016; Leij-Halfwerk et al., 2019; Koponen et al., 2021), and physical impairment (Distefano and Goodpaster, 2018). Aging also increases the risk of weight loss due to loss of appetite (van der Meij et al., 2017; Scheufele et al., 2023). Paradoxically, a one-fifth of people aged 65 years and above in Finland have a body mass index (BMI) exceeding 30 kg/m^2^, indicating overweight in older people (≥65 years) (Finnish institute for health and welfare, 2024; National Nutrition Council and Finnish Institute of Health and Welfare, 2020; National Research Council US Committee on Diet and Health, 1989). These demographic shifts present considerable challenges to both healthcare systems and the overall wellbeing of older people.

Normal aging involves changes in weight and body composition. Body weight generally increases until ages 60–70, followed by minimal to moderate weight loss until age 75 (Koster et al., 2010; Jackson et al., 2012). Afterwards, weight decline may become more pronounced. Similarly, fat mass increases with age but decreases slightly in older age (Koster et al., 2010; Jackson et al., 2012). Meanwhile, lean mass decreases from middle age onward (Koster et al., 2010; Jackson et al., 2012). These changes increase the risk for various adverse consequences such as functional impairment, frailty, falls, hospitalization, and mortality (Pilgrim et al., 2015; Distefano and Goodpaster, 2018).

The optimal weight for older people is less clear compared to younger adults. The World Health Organization defines a BMI of 18.5–24.9 as normal weight for adults (World Health Organization, 2024). However, a BMI between 25 and 30 kg/m^2^ has shown protective effects against mortality, and a range of 27.5–29.9 kg/m^2^ has been indicated as protective against comorbidity risk in older people (Pes et al., 2019). Notably, frailty modifies the U-shape association between BMI and mortality, suggesting that a higher BMI may protect frail older people from mortality (Watanabe. et al., 2024),. Finnish nutrition recommendations (National Nutrition Council and Finnish Institute of Health and Welfare, 2020) and the National Research Council (US) (National Research Council US Committee on Diet and Health, 1989) suggest a BMI range of 24–29 kg/m^2^ for normal weight in older people, supporting the idea of a higher optimal BMI for healthy aging.

Weight changes, not only being underweight or overweight, impact healthy aging. For instance, de Araujo et al. (2020) reported that older people experiencing weight loss had a higher incidence of comorbidities and hospitalizations, while those who gained weight reported poorer overall health. In addition, unintentional weight loss is associated with poor appetite, fewer teeth, and an increased risk of mortality, even among overweight and obese older people (De Stefani et al., 2018; Takehara et al., 2021; Scheufele et al., 2023). Factors such as female sex, depressive symptoms, polypharmacy, and chewing problems, which contribute to poor appetite (Scheufele et al., 2023), may also increase the risk of weight loss during aging.

Older family caregivers and their care recipients are at high risk for poor nutrition, including malnutrition and lower-than-recommended dietary intake, compared to community-dwelling older people without caregiving roles (Rullier et al., 2014; Rullier et al., 2013; Tombini et al., 2016; Leij-Halfwerk et al., 2019; Koponen et al., 2021). This increased vulnerability may also lead to an increased susceptibility to weight changes. Currently, research on obesity, weight loss, and weight gain among older family caregivers and care recipients is lacking. Similarly, there are no studies examining the impact of individually tailored nutritional guidance on weight changes or identifying factors influencing weight changes. Understanding the factors associated with weight loss or gain in older family caregivers and care recipients could enhance healthcare professionals’ better monitor and manage weight, providing valuable insights for tailored guidance.

This study aimed to identify prevalence of underweight (BMI <24 kg/m^2^) and overweight (BMI >29 kg/m^2^) among older family caregivers (≥60 years) and care recipients (≥65 years), along with identifying characteristic differences between underweight, normal weight and overweight. Furthermore, the study aimed to examine group differences and factors associated with weight change during individually tailored nutritional and oral health guidance.

## 2 Materials and methods

### 2.1 Study design and participants

The present study is a secondary analysis from the Lifestyle, Nutrition, and Oral Health in Caregivers (LENTO) study, a randomized, controlled, population-based trial involving older family caregivers (≥60 years of age) and care recipients (≥65 years of age) in Eastern Finland (Nykänen et al., 2021). The study adhered to the Declaration of Helsinki guidelines and received approval from the Hospital District of Northern Savo ethics committee (No. 171/2019). All participants provided written information consent, and the study was registered at ClinicalTrial.gov (NCT04003493).

**FIGURE 1:**
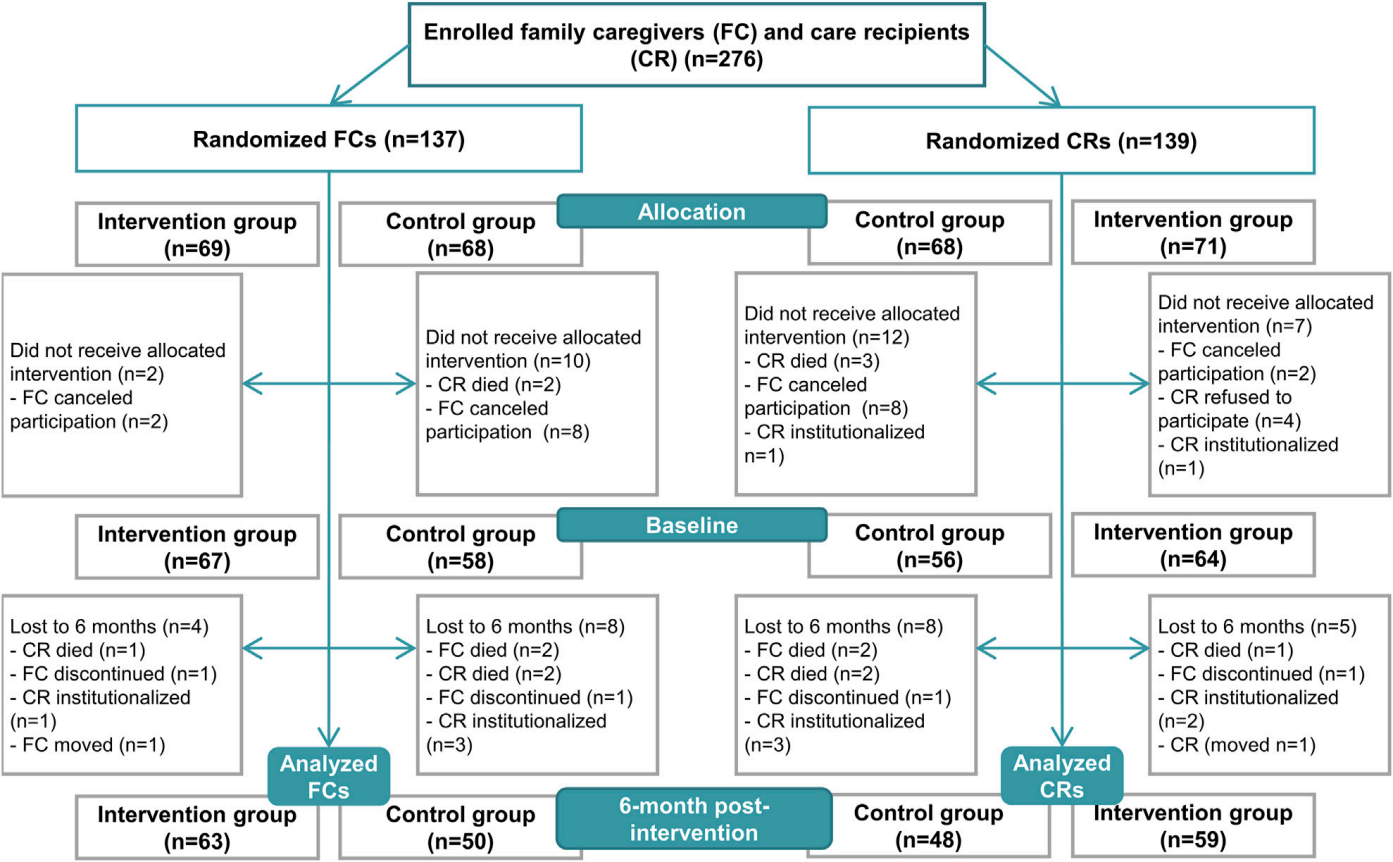
Study flow chart, modified from the study by Koponen et al., 2022.

Older family caregivers, along with their care recipients, residing in the town of Kuopio or the municipality of Vesanto were included in the study (Figure 1). Recruitments occurred between June 2019 and October 2019 in collaboration with the service managers for older people in municipalities, as previously reported (Koponen et al., 2021). The inclusion criteria for family caregivers included a valid care allowance from the municipality and a home-living care recipient aged 65 years or above. A care allowance provides benefits to the family caregiver such as a taxable fee and a 3-day leave per month. Family caregivers with care recipients receiving end-of-life care at the baseline were excluded. No other inclusion or exclusion criteria were stated. The study period extended from June 2019 to December 2020.

The sample size calculation was based on the effectiveness of the intervention on plasma albumin (P-Alb) concentration, aiming for a 20% difference between the intervention and the control group with a power of 0.80 and a *p*-value of 0.05. We have previously shown that individually tailored nutritional guidance is effective in older community-dwelling people based on serum albumin concentration (Nykänen et al., 2021). Consequently, a sample size of 128 (n = 64 per group) was calculated to demonstrate a statistically significant difference between the groups.

### 2.2 Study protocol

After enrollment, family caregivers along with their care recipients were randomly assigned to either the intervention or the control group using IBM SPSS Statistics software (v. 27, IBM Corp., Armonk, NY, USA), with randomization conducted without specific criteria. The allocation ratio was set at 1:1.

The LENTO study protocol was followed as described previously (Nykänen et al., 2021; Koponen et al., 2022). The participants underwent three home visits: two at baseline and one at the end of the 6-month intervention. Family caregivers provided interview details, while care recipients participated if able and willing. The baseline visits included a home visit by a study nurse followed by a joint visit by a clinical nutritionist and a dental hygienist a week later. The 6-month visit was conducted by the clinical nutritionist (between December 2019 and 16 March 2020). Due to the COVID-19 pandemic, some participants received the 6-month home visit by the study nurse for necessary measurements with their permission, and a phone interview from the clinical nutritionist (between 17 March 2020 and June 2020). Personal protective equipment and safety clearance were used during the home visits during the COVID-19 pandemic.

The intervention group received individualized nutritional and oral health care during two home visits: at baseline and at 2 months (Koponen et al., 2022). Nutritional guidance aligned with the National and Nordic Recommendations (Nordic Council of Ministers, 2012; National Nutrition Council, 2014), involving assessments of nutritional status, dietary intake, and diagnosed diseases. The clinical nutritionist guided family caregivers, on consuming five daily meals, achieving a protein intake of 1.2–1.4 g/kg body weight (BW)/d, achieving a fluid intake of at least 1 L/d, ensuring sufficient energy intake, and favoring sources of unsaturated fatty acids. Recommendations also included five daily servings of vegetables, fruits, and berries, with vitamin D supplements ranging from 10 to 20 μg/d based on dietary intake. Oral nutritional supplements and other dietary aids, for example, to supplement energy intake with vegetable oils, were suggested as needed. The dental hygienist guided family caregivers and care recipients on dental self-care practices, including teeth and mouth cleaning, addressing the perception of dry mouth, recommending regular dental examinations, and suggesting dental care services for those with acute needs. The oral health of family caregivers was integrated into individually tailored nutritional guidance. The control group did not receive interventions but were directed to health and dental care services if needed.

### 2.3 Measurements

The primary outcomes of the study were BMI and weight change during the 6-month intervention. Weight measurements were conducted by the clinical nutritionist for both family caregivers and care recipients, as well as height at the baseline. The measurements were taken using a calibrated portable weight scale and height measure to ensure accuracy and consistency across all participants.

During the baseline visit, the study nurse conducted comprehensive interviews with both family caregivers and care recipients, gathering essential background information, such as gender, age, relationship of family caregivers and care recipients, household’s net income, and years of education of family caregiver, and assessing comorbidities using modified Functional Comorbidity Index (FCI) (Groll et al., 2005; Tikkanen et al., 2012). The following diagnosed diseases were identified for the FCI: rheumatoid arthritis and other inflammatory connective tissue diseases; osteoporosis; diabetes type I or II; chronic asthma or chronic obstructive pulmonary disease; coronary artery disease; heart failure; myocardial infarction; stroke; depressive disorder; visual impairment; hearing impairment; dementia; and Parkinson’s disease. A higher value of FCI indicates a higher number of comorbidities. Simultaneously, information on medication use was recorded using medication lists, packages, and prescriptions. To complement this, non-fasting blood samples were collected, and concentrations of blood hemoglobin (B-Hb), plasma albumin (P-Alb), plasma prealbumin (P-Prealb), and plasma high-sensitivity C-reactive protein (P-hs-CRP) were analyzed using standard protocols at the Eastern Finland Laboratory Centre.

Additionally, the study nurse evaluated family caregivers’ cognitive function using the Mini-Mental State Examination (MMSE) (range 0–30, higher scores indicating better cognitive function) (Folstein et al., 1975) and depressive symptoms with the Geriatric Depression Scale (GDS-15) (range from 0 to 15, higher scores indicating higher number of depressive symptoms) (Yesavage and Sheikh, 1986). Family caregivers’ psychological distress and quality of life were assessed using General Health questionnaire (GHQ-12) (scores 0–12, higher scores indicating higher presence of psychological distress) (Goldberg, 1972) and the World Health Organization Quality of Life -brief version (WHOQOL-Bref) (range 0–130, higher scores indicating better quality of life) (The WHOQOL Group, 1998), respectively. Functional ability was assessed through activities of daily living (ADL) using the Barthel Index (range 0–100, higher scores indicating better functional ability) (Mahoney and Barthel, 1965) and instrumental activities of daily living (IADL) using the Lawton and Brody Scale (range 0–8, higher scores indicating better functional ability) (Lawton and Brody, 1969) from family caregivers. Additionally, family caregiver’s sense of coherence was evaluated with Sense of Coherence −13 (SOC-13) (range from 13 to 91, higher scores indicating better sense of coherence) (Antonovsky, 1987).

Frailty status was assessed from family caregivers with the abbreviated Comprehensive Geriatric Assessment (aCGA) scale, as previously described by Kiljunen et al. (2023), which is based on the full CGA detecting frailty in vulnerable older people (Overcash et al., 2005). The aCGA compounds 15 questions from MMSE (attention and calculation, reading, writing, and copying), ADL (bathing, transfer, and continence), IADL (shopping, preparing meals, housework, and laundry), and GDS-15 (emptiness, happiness, helplessness, and worthlessness) (Overcash et al., 2005). The family caregivers were divided into two groups (frailty and no frailty) by aCGA. Frailty was indicated with a positive score (≥1) at least in one domain (cognitive status, functional status, depression) of aCGA. The cut-off value for cognitive status was ≤6, for functional status it was ≥1, and for depression it was ≥2.

MMSE, GDS-15, GHQ-12, WHOQOL-Bref, ADL, IADL, SOC-13, and aCGA were specifically conducted for family caregivers, as they constituted the primary target group of the LENTO intervention study and were examined in more detail in the primary analysis.

In addition to these assessments, at the baseline visit the clinical nutritionist assessed nutritional status of family caregivers and care recipients using the Mini Nutritional Assessment (MNA) tool, including mid-arm and calf circumferences, a validated assessment for screening and assessing nutritional status of older people (≥65 years) (range 0–30, scores <17 indicating malnutrition, 17–23.5 indicating risk of malnutrition, and ≥24 indicating normal nutritional status) (Guigoz et al., 2002; Guigoz, 2006; Vellas et al., 2006). Moreover, the clinical nutritionist measured physical function of family caregivers and care recipients using a hand grip strength test (Saehan Hydraulic Hand Dynamometer) (Roberts et al., 2011) and 5-times chair stand test (Guralnik et al., 1994).

Dietary intake of family caregivers, including energy and nutrient intake, was assessed at the baseline by the clinical nutritionist using 3-day food records. The selection of a minimum 3 days for food record was based on its ability to capture usual food consumption, also in older population (Lührmann et al., 1999; Ortega et al., 2015). For those who had not maintained a food record, the clinical nutritionist performed a 24-h dietary recall. Dietary intake data were analyzed using AivoDiet software (v. 2.2.0.0, Aivodiet by Mashie, Turku, Finland), and compliance with nutrition recommendations, such Nordic Nutrition Recommendations (Nordic Council of Ministers, 2012) and National Nutrition Recommendations (National Nutrition Council, 2014), was evaluated.

The dental hygienist conducted a comprehensive clinical examination for both family caregivers and care recipients at the baseline encompassing factors such as the number of teeth and the use of removable dentures. Additionally, a thorough interview discovered perceptions of dry mouth, swallowing, and chewing issues. Participants provided responses on a four-point scale (0 = no problems, 1 = one problem, 2 = two problems, 3 = three problems). The inquiry process involved three key questions posed by the dental hygienist. Firstly, participants were asked “Do you have a feeling of dry mouth?,” with a response of “no” indicating no issue, while a response of “yes, sometimes” or “yes, continuously” was identified as one problem; “Can you chew hard or tough food, for example, rye bread, meat or apple?,” response of “without difficulties” indicated no problem, while responses “yes, but chewing is difficult” or “not at all” were identified as one problem; and “Can you eat dry bread or biscuit without drinking at the same time?,” a response “yes” indicated no problem, whereas a response of “no” indicated one problem.

### 2.4 Statistical analyses

An intention-to-treat approach was used in the statistical analyses. Baseline characteristics were summarized using means with standard deviations (SD) or number with percentages. The family caregivers and care recipients were categorized as underweight with BMI <24 kg/m^2^, normal weight with BMI 24 to 29, and overweight with BMI >29 based on Finnish nutritional recommendations for older people and recommendation of National Research Council (US) in Diet and Health (National Research Council US Committee on Diet and Health, 1989; National Nutrition Council and Finnish Institute of Health and Welfare, 2020).

Group differences, both intervention and control groups and when categorized by BMI at baseline, were analyzed using independent samples t-tests (two groups and normally distributed outcomes), Mann-Whitney U tests (two groups and non-normally distributed outcomes), ANOVAs (three groups and normally distributed outcomes), Kruskal–Wallis H test (three groups and non-normally distributed outcomes), Dunn’s test adjusted by the Bonferroni (pairwise comparison for three groups and non-normally distributed outcomes) or Pearson Chi-square test (categorized outcomes).

Difference between the groups (time-by-group interaction) in weight change and factors associated with the weight change during the 6-month intervention period were analyzed using a linear model of generalized estimating equations (GEE) (Twisk, 2004). Univariate GEE was used to analyze associations between independent variables (all factors described in the methods) and a dependent variable of weight change (kg). Significantly associated independent variables were selected for multivariate GEE analysis of weight change. In the GEEs, each one-unit increase in a factor predicts an x (B) increase/decrease in the dependent variable (weight change in kg) during the period, i.e., 6-month intervention. A significance level of <0.05 was set as the threshold for statistical significance. Multicollinearity between the variables was checked with the variance inflation factor (VIF). All data analyses were performed using IBM SPSS Statistics software (v. 27, IBM Corp., Armonk, NY).

## 3 Results

A total of 113 family caregivers and 107 care recipients were included in the analysis. The dropout rates during the intervention were 9.6% for family caregivers and 10.8% for care recipients (Figure 1). Due to the COVID-19 pandemic, the study nurse conducted the 6-month visit for 42 family caregivers and 37 care recipients (Koponen et al., 2022). Additionally, the clinical nutritionist conducted a 24-h dietary recall for 15 (13.3%) family caregivers at the baseline and 12 (10.6%) family caregivers at the 6-month time point.

### 3.1 Baseline characteristics

No differences in baseline characteristics were observed between the groups (Table 1; Supplementary Table 1; Koponen et al., 2022). Among the entire study population of family caregivers (n = 113), 73.5% were females, with a mean age of 74.3 (SD 7.1). Family caregivers’ households’ mean net income was 3,136 (SD 932) €/month, and their mean years of education was 11.0 (SD 3.3). The primary chronic diseases observed in family caregivers were rheumatoid arthritis or another connective tissue disease (37.5%) and diabetes, primarily type 2 (19.5%) (Supplementary Table 1; Koponen et al., 2022). Additionally, 36.3% of family caregivers were classified as overweight (BMI >29 kg/m^2^), while 18.6% were underweight (Table 1). According to MNA, 79.6% of family caregivers were well-nourished (≥24 scores), and 20.4% were at risk of malnutrition (17–23.5 scores) (Supplementary Table 1; Koponen et al., 2022). Moreover, 71.7% of family caregivers were identified as frail (Table 1). Notably, frailty was also prevalent among overweight family caregivers, with 68.3% exhibiting frailty status (not in Table). The mean number of teeth among family caregivers was 17.0 (SD 9.7), 44.2% used removable dentures (Table 1). On average, family caregivers reported 0.9 (SD 0.9) self-reported problems in their mouths.

**TABLE 1:** Baseline characteristics of the family caregivers and care recipients.

Characteristics	Family caregivers			Care recipients		
	Intervention group (n = 63)	Control group (n = 50)	*p*-value^a^	Intervention group (n = 59)	Control group (n = 48)	*p*-value^a^
	Mean ± SD	Mean ± SD		Mean ± SD	Mean ± SD	
Categorized BMI						
Underweight, <24 kg/m^2^, n (%)	10 (15.9)	11 (22.0)	0.255^c^	13 (24.5)^h^	8 (18.2)^i^	0.467^c^
Normal weight, 24–29 kg/m^2^, n (%)	26 (41.3)	25 (50.0)		20 (37.8)	22 (50.0)	
Obese, >29 kg/m^2^, n (%)	27 (42.9)	14 (28.0)		20 (37.8)	14 (31.8)	
Mid-arm circumference (cm)	33.3 (4.6)	31.7 (4.1)	0.056^b^	32.2 (5.4)^j^	31.7 (3.8)	0.877
Calf circumference (cm)	39.2 (4.1)	37.6 (3.5)	0.081	37.6 (5.0)^j^	36.1 (3.3)	0.063^b^
P-hs-CRP (g/L)	2.8 (5.0)	1.9 (2.3)	0.230	3.2 (6.1)	3.8 (2.0)	0.748
Frail by aCGA, n (%)	49 (77.8)	32 (64.0)	0.106^c^			
aCGA domains						
Cognitive status, n (%)^d^	27 (42.9)	18 (36.0)	0.460^c^			
Functional status, n (%)^e^	16 (25.4)	14 (28.0)	0.756^c^			
Depression, n (%)^f^	28 (44.4)	18 (36.0)	0.364^c^			
Hand grip strength (kg)	25.2 (8.7)	23.7 (7.1)	0.569	20.7 (7.5)^k^	21.9 (8.5)^l^	0.494^b^
Chair stand test (s)	13.4 (4.7)^g^	13.0 (4.0)	0.691	19.6 (5.1)^l^	20.0 (5.1)^m^	0.768^b^
SOC-13	62.2 (6.5)	61.1 (7.0)	0.285			
Number of teeth, n (%)	17.0 (9.8)	17.1 (9.7)	0.830	13.6 (9.9)	12.8 (9.4)	0.646
Dentures, yes, n (%)	30 (47.6)	20 (40.0)	0.426^c^	29 (53.7)	24 (53.3)	0.971^c^
Sel-reported problems in mouth	0.9 (1.0)	0.8 (0.8)	0.983	1.6 (0.9)	1.3 (1.1)	0.161

SD, standard deviation; BMI, body mass index, P-hs-CRP, plasma high-sensitivity C-reactive protein, aCGA, abbreviated comprehensive geriatric assessment; SOC-13, sense of coherence.

^a^ Difference between groups with Mann-Whitney’s U test (non-normally distributed outcomes).

^b^ Difference between groups with independent samples T-test (normally distributed outcomes).

^c^ Difference between groups with Pearson Chi-square.

^d^ aCGA, domain cognitive status: attention and calculation (Mini Mental State Examination), reading (Mini Mental State Examination), writing (Mini Mental State Examination), copying (Mini Mental State Examination); with a cut-off maximum ≤6.

^e^ aCGA, domain functional status: bathing (Barthel Index), transfer (Barthel Index), continence (Barthel Index), shopping (Lawton and Brody scale), preparing meals (Lawton and Brody scale), housework (Lawton and Brody scale), laundry (Lawton and Brody scale); with a cut-off maximum ≥1.

^f^ aCGA, domain depression: emptiness (Geriatric Depression Scale), happiness (Geriatric Depression Scale), helplessness (Geriatric Depression Scale), worthlessness (Geriatric Depression Scale); with a cut-off maximum ≥2.

^g^ n = 62.

^h^ n = 53.

^i^ n = 44.

^j^ n = 58.

^k^ n = 59.

^l^ n = 48.

^m^ n = 21.

The mean energy intake of family caregivers was 1711 kcal/d, with 45.0 E% delivered from carbohydrates, 16.4 E% from protein, and 34.5 E% from fat (Koponen et al., 2022). Their mean protein intake was 0.97 g/kg BW/d, with 21.2% achieving the recommended intake of 1.2 g/kg BW/d for older people (National Nutrition Council and Finnish Institute of Health and Welfare, 2020). At the baseline, 7.1% reported a moderate, and 0.9% severe decrease in dietary intake over the past 3 months (not in Table).

In the entire study population of care recipients (n = 107), 33.6% were females, and their mean age was 79.3 (SD 7.9) in the whole study population (Supplementary Table 1; Koponen et al., 2022). The prevalent chronic diseases among care recipients were dementia (57.9%) and diabetes, mainly type 2 (33.6%) (Supplementary Table 1; Koponen et al., 2022). BMI indicated that 21.6% of care recipients were underweight (BMI <24 kg/m^2^), while 35.1% were classified as overweight (BMI >29 kg/m^2^). The proportions of well-nourished, at risk of malnutrition, and malnourished care recipients were 30.8%, 62.6%, and 6.5%, respectively (Supplementary Table 1; Koponen et al., 2022). The mean number of teeth in care recipients was 13.3 (SD 9.7), with 53.5% of them using removable dentures (Table 1). Care recipients reported an average of 1.5 (SD 1.0) self-reported problems in mouth.

### 3.2 Differences between underweight, normal weight, and overweight family caregivers and care recipients at baseline

Table 2 illustrates the differences between BMI categories. Notably, overweight family caregivers were found to be significantly younger than their normal weight counterparts (*p* = 0.007). Moreover, both mid-arm and calf circumferences increased significantly based on BMI categorization (*p* < 0.001), with underweight family caregivers having the smallest mid-arm and calf circumferences, and overweight having the largest. Furthermore, overweight family caregivers had significantly higher P-Prealb concentration compared to underweight or normal weight family caregivers (*p* < 0.001 and 0.039, respectively). However, no significant differences were observed in MNA scores, frailty status, dietary intake, or any other outcomes across BMI categories (underweight vs. normal weight vs. overweight) (not in Table).

**TABLE 2:** Baseline characteristics of older family caregivers (n = 113) and care recipients (n = 97) according to body mass index (BMI) categories.

Characteristics	Family caregivers						
	Underweight BMI <24 kg/m^2^ (n = 21)	Normal weight BMI 24–29 kg/m^2^ (n = 51)	Overweight BMI >29 kg/m^2^ (n = 41)	*p*-value^a^	*p*-value^c^ Underweight – Normal weight	*p*-value^c^ Underweight – Overweight	*p*-value^c^ Normal weight – Overweight
Age (y)	73.9 (9.2)	76.0 (6.1)	72.3 (6.8)	**0.041**	0.248	0.443	**0.007**
Mid-arm circumference (cm)	27.3 (2.3)	31.2 (1.9)	37.0 (3.3)	**<0.001** ^b^	**0.001** ^d^	**<0.001** ^d^	**<0.001** ^d^
Calf circumference (cm)	34.4 (2.7)	37.4 (2.2)	42.0 (3.2)	**<0.001** ^b^	**0.007** ^d^	**<0.001** ^d^	**<0.001** ^d^
P-Prealb (g/L)	0.22 (0.04)	0.24 (0.05)	0.26 (0.04)	**0.004**	0.099	**<0.001**	**0.039**

Characteristics	Care recipients						
	Underweight BMI <24 kg/m^2^ (n = 21)	Normal weight BMI 24–29 kg/m^2^ (n = 42)	Overweight BMI >29 kg/m^2^ (n = 34)	*p*-value	*p*-value^c^ Underweight – Normal weight	*p*-value^c^ Underweight – Overweight	*p*-value^c^ Normal weight – Overweight
Number of medications	6.6 (2.8)	8.0 (3.8)	11.2 (4.3)	**<0.001** ^b^	0.524	**<0.001**	**0.005**
Mid-arm circumference (cm)	27.3 (2.3)	31.4 (2.1)	35.6 (4.9)	**<0.001**	**<0.001**	**<0.001**	**<0.001**
Calf circumference (cm)	32.9 (2.3)	36.5 (2.7)	40.7 (4.2)	**<0.001**	**<0.001**	**<0.001**	**<0.001**
MNA scores	20.3 (3.1)	22.8 (2.7)	22.5 (3.1)	**0.009** ^b^	**0.007**	0.055	1.000
P-hs-CRP (g/L)^e^	1.1 (1.3)	3.1 (5.7)	4.4 (8.9)	**0.048** ^b^	0.097	0.064	1.000
Hand grip strength (kg)^f^	17.9 (6.4)	21.3 (6.9)	23.6 (9.6)	**0.043**	0.068	**0.022**	0.262
Chair stand test (s)^g^	18.5 (3.7)	22.0 (6.1)	17.9 (3.0)	**0.045**	0.087	0.650	**0.039**
Number of teeth^h^	17.4 (9.1)	14.8 (9.0)	9.1 (9.8)	**0.005** ^b^	1.000	**0.008**	**0.040**

BMI, body mass index, P-Prealb = plasma prealbumin concentration, MNA, mini nutritional assessment, P-hs-CRP, plasma high-sensitivity C-reactive protein. Bold values denote statistical significance at the p < 0.05 level.

^a^ Difference between groups with one-way ANOVA (normally distributed outcomes).

^b^ Difference between groups with Kruskal–Wallis H Test (non-normally distributed outcomes).

^c^ Difference between groups independent samples t-test (normally distributed outcomes).

^d^ Difference between groups with Dunn’s Test adjusted by the Bonferroni (non-normally distributed outcomes).

^e^ Underweight n = 21, normal weight n = 41, obese n = 32.

^f^ Underweight n = 20, normal weight n = 37, obese n = 31.

^g^ Underweight n = 12, normal weight n = 18, obese n = 12.

^h^ Underweight n = 21, normal weight n = 39, obese n = 34.

On the contrary, overweight care recipients exhibited a higher number of medications compared to both underweight and normal weight care recipients (<0.001 and *p* = 0.005, respectively) (Table 2). The trend in mid-arm and calf circumferences among care recipients mirrored that observed in family caregivers. Conversely, underweight care recipients displayed the lowest MNA scores, significantly lower than those of normal weight care recipients (*p* = 0.007). Furthermore, a significant difference was noted between the BMI categories in P-hs-CRP (0.048); however, upon closer examination using an independent samples t-test to analyze differences between two BMI categories, no significant differences were found. In terms of functional capacity, underweight care recipients exhibited significantly lower hand grip strength compared to overweight care recipients (*p* = 0.022), and normal weight care recipients demonstrated significantly longer time in the 5-times chair stand test compared to overweight care recipients (*p* = 0.039). Additionally, both underweight and normal weight care recipients had a significantly higher number of teeth compared to overweight care recipients (*p* = 0.008 and *p* = 0.040, respectively).

### 3.3 Weight changes

**FIGURE 2:**
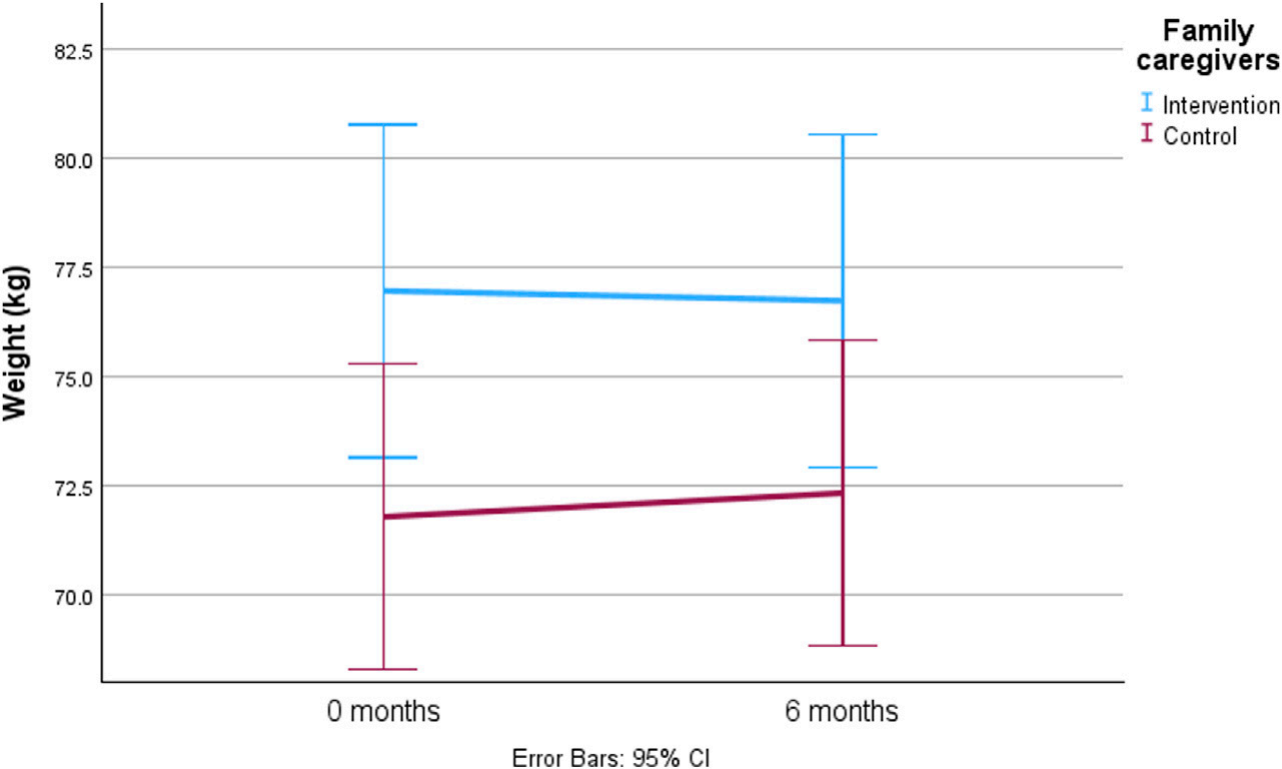
The predicted mean value with 95% confidence intervals of weight change among older family caregivers during the 6-month intervention by generalized estimating equations (GEEs) adjusted with time-by-group interaction, sex, age, change in dietary intake, frailty, blood hemoglobin (B-Hb), plasma prealbumin (P-Prealb), mid-arm circumference, calf circumference, hand grip strength, and protein intake.

Table 3; Figure 2 show that there was no significant difference (time-by-group interaction) in the weight of family caregivers between the intervention and control groups over the 6-month intervention period. Baseline factors significantly associated with the change in weight in older family caregivers are represented in Table 3, as determined through both univariate and multivariate analyses. Univariate analyses demonstrated that baseline factors such as female sex (*p* = 0.034), older age (*p* = 0.013), severe decrease in dietary intake over the past 3 months (*p* < 0.001), frailty (*p* = 0.041), lower B-H concentration (*p* = 0.009), lower P-Prealb concentration (*p* < 0.001), smaller mid-arm circumference (*p* < 0.001), smaller calf circumference (*p* < 0.001), weaker hand grip strength (*p* < 0.001), and protein intake ≥1.2 g/kg BW/d (*p* < 0.001) were independently associated with weight loss during the 6-month intervention period in older family caregivers (Table 3). No other baseline characteristics described in the methods were associated with the weight change of family caregivers during the intervention. The multivariate analysis identified female sex (*p* < 0.001), no decrease in food intake over the past 3 months compared to severe decrease (*p* = 0.001), frailty (*p* = 0.040), smaller mid-arm circumference (*p* < 0.001), and smaller calf circumference to be associated with weight loss in older family caregivers during the 6-month intervention period (Table 3).

**TABLE 3:** Associated baseline factors of weight change (kg) during the 6-month intervention in older family caregivers by univariate (n = 116) and multivariate (n = 114) generalized estimating equations (GEEs).

	Univariate			Multivariate		
	Weight change (kg)			Weight change (kg)		
	B (SE)	95% CI	*p*-value	B (SE)	95% CI	*p*-value
Time x group						0.491
Sex, ref. male	−6.52 (3.08)	−12.56, −0.48	**0.034**	−10.41 (1.23)	−12.82, −8.00	**<0.001**
Age, y	−0.55 (0.22)	−0.98, −0.12	**0.013**			
Change in dietary intake, ref. no decrease in food intake			**<0.001**			**0.004**
Moderate decrease in food intake	−2.84 (7.39)	−17.33, 11.64	0.700	1.81 (3.25)	−4.56, 8.19	0.577
Severe decrease in food intake	−11.43 (1.70)	−14.76, −8.10	**<0.001**	5.52 (1.67)	2.24, 8.80	**0.001**
Frailty, ref. no frail	−7.01 (0.85)	−13.72, −0.29	**0.041**	−2.34 (1.14)	−4.58, −0.10	**0.040**
B-Hb, g/L	0.32 (0.12)	0.08, 0.56	**0.009**			
P-Prealb, g/L	132.94 (32.28)	69.66, 196.22	**<0.001**			
Mid-arm circumference, cm	2.86 (0.19)	2.48, 3.23	**<0.001**	1.77 (0.22)	0.22, 1.33	**<0.001**
Calf circumference, cm	3.26 (0.25)	2.76, 3.75	**<0.001**	1.56 (0.23)	0.23, 1.12	**<0.001**
Hand grip strength, kg	0.67 (0.18)	0.31, 1.02	**<0.001**			
Protein recommendation, ref. <1.2 g/kg BW/d	−13.88 (2.78)	−19.34, −8.42	**<0.001**			

B-Hb = blood hemoglobin, P-Prealb = plasma prealbumin concentration, BW, body weight. Bold values denote statistical significance at the p < 0.05 level.

^a^ n = 114.

**FIGURE 3:**
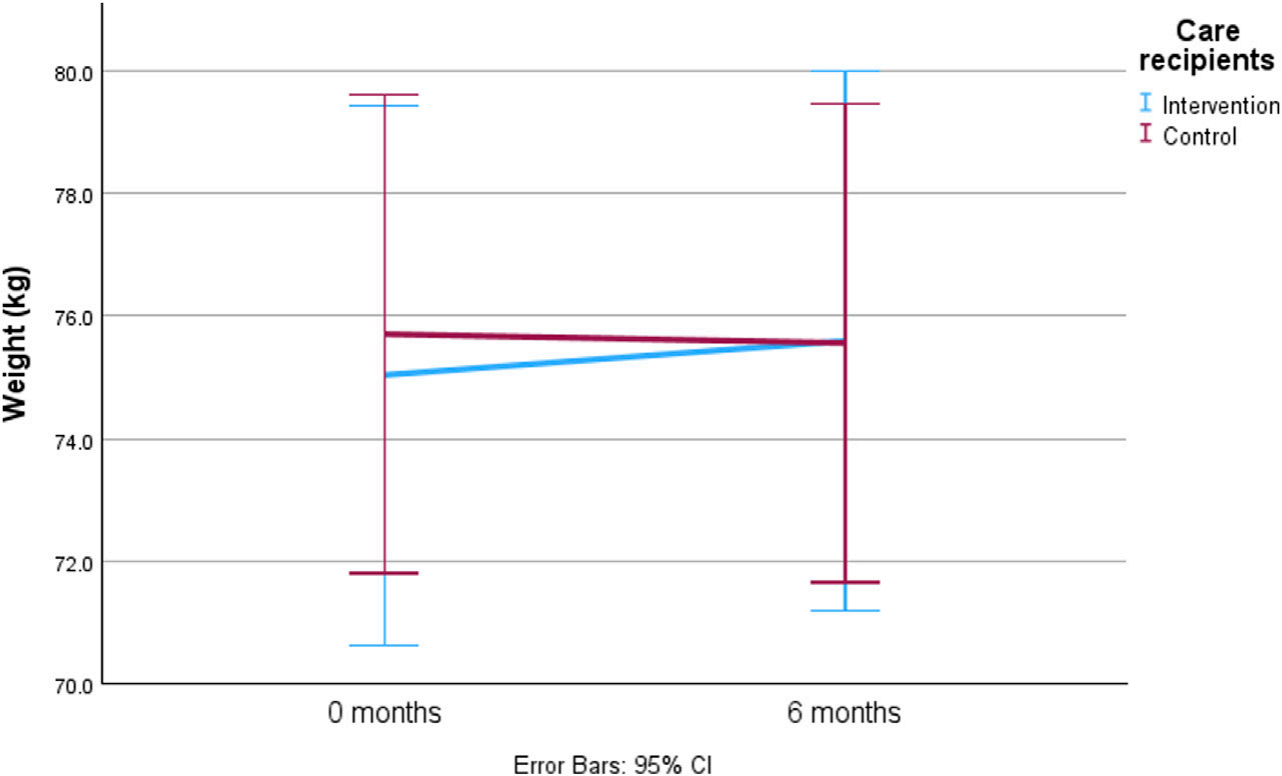
The predicted mean value with 95% confidence intervals for weight change among older care recipients during the 6-month intervention by generalized estimating equations (GEEs) adjusted with time-by-group interaction, sex, sex of family caregiver, age, number of medications, Mini Nutritional Assessment (MNA) scores, change in dietary intake, plasma albumin (P-Alb), plasma high-sensitivity C-reactive protein, mid-arm circumference, calf circumference, hand grip strength, and quality of life of family caregiver.

There was no significant between-group difference (time-by-group interaction) in the weight change of care recipients during the 6-month intervention period (Table 4; Figure 3). Baseline factors significantly associated with the weight change in older care recipients are represented in Table 4. Univariate analyses indicated that factors such as female sex of care recipient (*p* = 0.001), male sex of family caregiver (*p* = 0.002), older age (*p* = 0.017), lower number of medications (*p* < 0.001), lower MNA scores (*p* = 0.034), moderate decrease in dietary intake over the past 3 months (*p* < 0.001), higher P-Alb concentration (*p* = 0.025), lower P-hs-CRP concentration (*p* = 0.11), smaller mid-arm circumference (*p* < 0.001), smaller calf circumference (*p* < 0.001), weaker hand grip strength (*p* < 0.001), and lower WHOQOL-Bref scores of family caregiver (*p* = 0.014) were independently associated with weight loss in older care recipients during the 6-month intervention period (Table 4). No other baseline characteristics of care recipients and family caregivers described in the methods were associated with the weight change of care recipients during the intervention. In the multivariate analysis, female sex of care recipient (*p* < 0.001), and smaller mid-arm circumference (*p* < 0.001) and calf circumference (*p* < 0.001) were associated with weight loss in older care recipients during the 6-month intervention period (Table 4).

**TABLE 4:** Associated baseline factors of weight change (kg) during the 6-month intervention in older care recipients by univariate (n = 97) and multivariate (n = 86) generalized estimating equations (GEEs).

	Univariate			Multivariate		
	Weight change (kg)			Weight change (kg)		
	B (SE)	95% CI	*p*-value	B (SE)	95% CI	*p*-value
Time x group						0.706
Sex, ref. male	−11.91 (3.70)	−19.17, −4.66	**0.001**	−12.49 (2.45)	−17.30, −7.69	**<0.001**
Sex of family caregiver, ref. male	12.54 (3.97)	4.77, 20.32	**0.002**			
Age, y	−0.49 (0.21)	−0.89, −0.09	**0.017**			
Number of medications	1.75 (0.44)	0.90, 2.61	**<0.001**			
MNA scores	1.41 (0.66)	0.11, 2.71	**0.034**			
Change in dietary intake, ref. no decrease in food intake			**0.001**			
Moderate decrease in food intake	−11.65 (3.31)	−18.15, −5.16	**<0.001**			
Severe decrease in food intake	10.24 (17.53)	−24.12, 44.60	0.559			
P-Alb 0 months, g/L^a^	−1.30 (0.58)	−2.43, −0.16	**0.025**			
P-hs-CRP 0 months, g/L^b^	0.87 (0.34)	0.20, 1.53	**0.011**			
Mid-arm circumference, cm	3.43 (0.25)	2.94, 3.91	**<0.001**	2.56 (0.28)	2.01, 3.12	**<0.001**
Calf circumference, cm	3.12 (0.32)	2.50, 3.74	**<0.001**	1.03 (0.21)	0.63, 1.43	**<0.001**
Hand grip strength, kg^c^	0.94 (0.23)	0.49, 1.39	**<0.001**			
WHOQOL-Bref scores of family caregiver	0.30 (0.12)	0.06, 0.55	**0.014**			

MNA, mini nutritional assessment, P-Alb = plasma albumin concentration, P-hs-CRP, plasma high-sensitivity C-reactive protein, WHOQOL-Bref = World Health Organization Quality of Life – brief version. Bold values denote statistical significance at the p < 0.05 level.

^a^ n = 95.

^b^ n = 94.

^c^ n = 89.

## 4 Discussion

The present study observed a high prevalence of overweight and underweight among both family caregivers and care recipients. Overweight family caregivers tended to be younger and had greater mid-arm and calf circumference, and higher P-Prealb concentration. Among overweight care recipients, there was a significantly higher use of medications, along with greater mid-arm and calf circumference, stronger hand grip strength, faster 5-times chair stand test, and fewer teeth. In contrast, underweight care recipients had significantly lower MNA scores compared to normal weight care recipients. No significant changes in weight were observed in either family caregivers or care recipients. During the intervention, frailty and female sex were associated with weight loss, while greater mid-arm and calf circumference were associated with weigh gain in family caregivers. Similarly, among older care recipients, female sex was associated with weight loss, while greater mid-arm and calf circumference were associated with weight gain.

The prevalence of overweight in the present study was high among both family caregivers (36%) and care recipients (35%). These figures are significantly higher than those for the Finnish older population, according to the Healthy Finland Survey (Finnish institute for health and welfare, 2024), where 21% of the population aged 65 and above had a BMI greater than or equal to 30 kg/m^2^. The Healthy Finland survey uses a higher threshold for overweight, which may partly explain the large difference in the number of overweight family caregivers and care recipients. Furthermore, a noteworthy observation is that approximately one-fifth of both family caregivers and care recipients were classified as underweight in the present study. This prevalence of both overweight and underweight is alarming for their health, given that the risk of comorbidity and mortality increases with both lower and higher BMI (Pes et al., 2019). The reasons for this population’s higher prevalence of overweight cannot be determined from the present study; the status of the family caregiver and care recipient may partly explain this. Further studies comparing these differences are needed.

In the present study, the age of the family caregivers varied across BMI categories, with overweight caregivers being significantly younger than their normal weight counterparts. This finding suggests that BMI may increase with age among family caregivers, as indicated by previous research (Koster et al., 2010; Jackson et al., 2012). However, in the present study, age did not significantly influence weight changes in either group. Thus, the present result imply that age alone cannot predict weight loss or weight gain in this population, contrary to earlier studies involving older people (Yano et al., 2023). A longer follow-up time may be needed to observe these trends more accurately.

The present study reveals associations between greater mid-arm and calf circumference with higher BMI and weight gain in both family caregivers and care recipients. Guo et al. (2021) reported that mid-arm and calf circumference decline more rapidly than BMI due to aging. Lower mid-arm and calf circumference in underweight family caregivers and care recipients may particularly indicate a decline in muscle mass and further physical function (Sanchez et al., 2011; Asai et al., 2019; Liu et al., 2023). These findings suggest that mid-arm and calf circumference measurements could serve as convenient indicators for identifying underweight family caregivers and care recipients and those at higher risk for weight and muscle loss in clinical assessments.

The present study found that the concentration of P-Prealb was higher in overweight family caregivers compared to those who were underweight and normal weight. This finding aligns with Kobayashi et al. (2023), who also reported a similar association between BMI and serum albumin levels. This suggests that individuals with higher BMI may have a better nutritional status. However, P-Prealb can be influenced by factors such as inflammation and hydration (Keller, 2019; Evans et al., 2021). Therefore, caution is needed when interpreting this result.

Among older care recipients, those classified as overweight had a higher medication count compared to their underweight and normal weight counterparts, which aligns with earlier evidence (Assari et al., 2019). This finding highlights the importance of early identification of care recipients at risk of becoming overweight in old age to prevent associated adverse effects that can complicate caregiving.

Overweight care recipients exhibited better physical performance and better hand grip strength than underweight care recipients, and they completed the 5-times chair stand test faster than those of normal weight. Improved physical performance is known to have a positive impact on health, helping to protect against frailty (Jeoung and Lee, 2015). A higher BMI, which can protect frail older adults from mortality (Watanabe et al., 2024), may also help prevent physical decline due to better muscle status. However, conflicting findings exist. Tsai and Chang (2017) reported that a high BMI increases the risk for functional decline, even in those with good baseline functional ability. Therefore, a high BMI may not reliably predict the maintenance of good functioning in older people over time.

Overweight care recipients had fewer teeth compared to their underweight and normal weight counterparts. This finding aligns with previous research by Hayashi et al. (2022). The reduced number of teeth may be due to a higher prevalence of periodontal disease or dental caries, often linked to sugar consumption (Wood et al., 2003; Bernabé et al., 2014). Conversely, having fewer teeth may increase the risk for malnutrition, although evidence on this is conflicting (Algra et al., 2021). Identifying older people who are both obese and have fewer teeth is crucial for implementing targeted interventions to maintain or improve their health.

Underweight care recipients in the present study had a significantly lower MNA scores compared to normal weight care recipients. This finding aligns with earlier research highlighting the adverse effects of being underweight status on the nutritional wellbeing of older people (Burman et al., 2015). It underscores the importance of maintaining a BMI at least 24 kg/m^2^ during older age.

The present study found no differences in weight between family caregivers and care recipients in the intervention and control groups during the 6-month intervention. The study aimed to improve nutrition for older family caregivers through individually tailored nutritional guidance, including maintaining their weight. Maintaining a stable weight is generally beneficial for health, also in older age. In the present study, both groups maintained their weight throughout the 6-month intervention. However, it is important to identify individuals at high risk for unfavorable weight changes within these subgroups. Furthermore, some family caregivers and their care recipients experienced weight loss. This could be attributed to the significant caregiving responsibilities of older family caregivers, which may have impacted their ability to consistently provide nutritious meals for themselves and their care recipients.

The findings suggest that female sex in both study subgroups, namely, family caregivers and care recipients, was associated with weight loss. This aligns with existing knowledge that older females are susceptible to “anorexia of aging”, characterized by declining appetite and an increased risk of weight loss (Pilgrim et al., 2015). Although self-reported severe decreases in food intake over the past 3 months at baseline predicted weight loss in univariate analyses, this effect did not persist in the multivariate analysis. Moreover, other nutritional factors did not show significant association with weight change, emphasizing the need for a more accurate evaluation of dietary intake and appetite effects on weight change in older people.

The study underscores the significance of frailty status among family caregivers as a significant factor in weight loss, aligning with earlier findings (Crow et al., 2020). Frailty, which is associated to malnutrition, cognitive decline, physical disability, depression, morbidity, hospitalization, and mortality (De Breij et al., 2021; Cohen et al., 2023; Kiljunen et al., 2023), highlights the need for healthcare professionals to identify and intervene to prevent weight loss. Family caregivers at risk of frailty can benefit from prevention and treatment through various effective approaches, including physical training, cognitive training, and nutritional interventions (Cohen et al., 2023). It should be noted that the aCGA used to measure frailty in the present study includes not only physical aspects but also cognitive status and depression domains in addition to functional status. This highlights that physical limitations alone do not explain weight loss in the context of frailty. It also underscores the impact of psychological and cognitive status on weight loss.

Notably, characteristics such as income, education, cognition, depression, physical ability (ADL and IADL), quality of life, sense of coherence, and oral health did not show associations with weight change in family caregivers. Therefore, no additional specific factors were identified for this nutritionally vulnerable subgroup of older people beyond well-known factors such as female sex, frailty, physical function, and anthropometrics. Furthermore, family caregivers’ characteristics did not associate with weight change in their care recipients in multivariate analysis. However, it is noteworthy that univariate analyses suggested associations between male sex and quality of life of family caregivers, and weight change of their care recipients. Therefore, further research is needed to identify the role of these specific characteristics of family caregivers that may explain weight loss in older care recipients, as maintaining weight in older age is important for healthy aging.

In practical implementations for older family caregivers and care recipients, regular body weighing and monitoring for changes in weight and factors contributing to weight loss are essential. A comprehensive approach involving regular health inspections for both family caregivers and care recipients plays a vital role in successful caregiving. However, further research is needed to refine these practices. This approach is crucial for managing healthy body weight and addressing the adverse effects of overweight, weight loss, and weight gain. For example, healthcare providers should follow Finnish nutrition recommendations for older people (National Nutrition Council and Finnish Institute of Health and Welfare, 2020), advocating weighing at least once a month or more frequently if needed, to detect unfavorable weight changes promptly in both family caregivers and care recipients.

Strengths of the study include its multiprofessional approach, population-based design, and use of validated methods. Furthermore, data collection involved trained professionals, including the study nurse, clinical nutritionist, and dental hygienist. However, a limitation is that participation in the intervention study may have been perceived as burdensome by some family caregivers, and potentially excluding the most stressed caregivers and limiting the sample size. Furthermore, the optimal BMI for older people remains unclear globally, with BMI thresholds of <24 for underweight and >29 for overweight not standardized universally. The study also had limitations in assessing care recipients comprehensively compared to their family caregivers, with various social and functional characteristics missing. The main focus of the LENTO intervention study was on family caregivers, which aimed to reduce study burden and non-participation, further limiting the sample size. However, the study did consider family caregivers characteristics as potential factors influencing weight changes in their care recipients. Notably, caregiver burden was not included in the study protocol, which could have provided valuable insights in the analyses.

In conclusion, being overweight is a prevalent condition among older family caregivers and care recipients. Overweight was more common in younger family caregivers and in care recipients with a higher number of medications, better physical function, and fewer teeth. Underweight care recipients had lower MNA scores. Female sex was associated with weight loss in both older family caregivers and care recipients, and frailty was associated with weight loss in caregivers. However, the characteristics of family caregivers did not explain the weight loss of their care recipients.

## Data Availability

The original contributions presented in the study are included in the article/Supplementary Material, further inquiries can be directed to the corresponding author.

## Abbreviations

aCGA, abbreviated Comprehensive Geriatric Assessment; ADL, activities of daily living; B-Hb, blood hemoglobin concentration; BMI, body mass index; BW, body weight; FCI, Functional Comorbidity Index; GDS-15, Geriatric Depression Scale; GEEs, Generalized Estimating Equations; GHQ-12, General Health Questionnaire; IADL, instrumental activities of daily living; LENTO, Lifestyle, Nutrition, and Oral Health in Caregivers study; MMSE, Mini-Mental State Examination; MNA, Mini Nutritional Assessment; P-Alb, plasma albumin concentration; P-hs-CRP, plasma high-sensitivity C-reactive protein; P-Prealb, plasma prealbumin concentration; SD, standard deviation; SOC-13, Sense of Coherence −13; VIF, variance inflation factor; WHOQOL-Bref, World Health Organization Quality of Life -brief version.
