# Supplementary material for: Underweight, overweight, and weight change in older family caregivers and their care recipients: longitudinal evidence from a randomized controlled trial
Source: Front Aging. 2024 Aug 15;5:1376825. doi: 10.3389/fragi.2024.1376825 (PMC11358125; doi:10.3389/fragi.2024.1376825)
Supplement: Supplementary file 1 [file DataSheet1.pdf]

Supplementary table 1 Baseline characteristics of the family caregivers and care recipients, previously published by Koponen et al. (2022).

Characteristics	Family caregivers			Care recipients		
	Intervention group (n=63) Mean ± SD	Control group (n=50) Mean ± SD	p-value [a]	Intervention group n=59 Mean ± SD	Control group n=48 Mean ± SD	p-value [a]
Females, n (%)	45 (71.4)	38 (76.0)	0.585 [c]	22 (37.3)	14 (29.2)	0.377 [c]
Age (y)	74.5 (6.4)	74.0 (8.0)	0.704 [b]	79.6 (7.9)	78.9 (7.9)	0.653 [b]
FCI	2.0 (1.6)	2.0 (1.4)	0.631	3.5 (2.0)	3.5 (2.0)	0.819
Number of medications	5.6 (4.1)	5.1 (3.3)	0.506	8.5 (4.2)	8.8 (4.5)	0.786 [b]
BMI (kg/m^2)	29.3 (6.3)	27.4 (4.7)	0.113	29.0 (7.0) [g]	27.6 (4.3) [h]	0.723
MNA scores	25.4 (1.9)	25.5 (2.0)	0.984	24.5 (0.5)	21.9 (3.0)	0.625 [b]
Normal nutritional status	51 (81.0)	39 (78.0)	0.699 [c]	17 (28.8)	16 (33.3)	0.238 [c]
Risk for malnutrition	12 (19.0)	11 (22.0)		36 (61.0)	31 (64.6)	
Malnutrition				6 (10.3)	1 (2.1)	
B-Hb (g/L)	135.8 (10.5) [d]	134.7 (10.5)	0.607 [b]	136.4 (13.4) [i]	134.7 (15.4) [j]	0.973
P-Alb (g/L)	37.5 (2.3)	37.6 (2.4) [e]	0.948	34.8 (3.5)	34.0 (3.4) [k]	0.316
P-Prealb (g/L)	0.25 (0.04)	0.24 (0.05)	0.431 [b]	0.23 (0.04) [l]	0.23 (0.05) [k]	0.675 [b]
MMSE	26.1 (3.2)	26.9 (2.8)	0.552			
GDS-15	3.1 (2.3)	2.8 (2.7)	0.309			
GHQ-12	2.8 (2.8)	2.2 (3.1)	0.055			
WHOQOL-Bref	99.1 (11.4) [f]	98.6 (12.5)	0.834 [b]			
ADL	97.9 (3.3)	99.3 (3.7)	0.606			
IADL	6.8 (0.6)	6.9 (0.5)	0.199			

SD = standard deviation, FCI = functional comorbidity index, BMI = body mass index, MNA = Mini Nutritional Assessment, B-Hb = blood hemoglobin concentration, P-Alb = plasma albumin concentration, P-Prealb = plasma prealbumin concentration, MMSE = Mini-Mental State Examination, GDS-15 = Geriatric Depression Scale, GHQ-12 = General Health Questionnaire, WHOQOL-Bref = World Health Organization Quality of Life Brief version, ADL = activities of daily living by Barthel Index, IADL = instrumental activities of daily living by Lawton & Brody Scale.

[a] Difference between groups with Mann-Whitney's U test (non-normally distributed outcomes)

[b] Difference between groups with independent samples T-test (normally distributed outcomes)

[c] Difference between groups with Pearson Chi-square

[d] n=61, [e] n=49, [f] n=62, [g] n=53, [h] n=44, [i] n=45, [j] n=46, [k] n=57, [l] n=56
